# MGL Receptor and Immunity: When the Ligand Can Make the Difference

**DOI:** 10.1155/2015/450695

**Published:** 2015-12-29

**Authors:** Ilaria Grazia Zizzari, Chiara Napoletano, Federico Battisti, Hassan Rahimi, Salvatore Caponnetto, Luca Pierelli, Marianna Nuti, Aurelia Rughetti

**Affiliations:** Department of Experimental Medicine, “Sapienza” University, Viale Regina Elena 324, 00161 Rome, Italy

## Abstract

C-type lectin receptors (CLRs) on antigen-presenting cells (APCs) facilitate uptake of carbohydrate antigens for antigen presentation, modulating the immune response in infection, homeostasis, autoimmunity, allergy, and cancer. In this review, we focus on the role of the macrophage galactose type C-type lectin (MGL) in the immune response against self-antigens, pathogens, and tumor associated antigens (TAA). MGL is a CLR exclusively expressed by dendritic cells (DCs) and activated macrophages (MØs), able to recognize terminal GalNAc residues, including the sialylated and nonsialylated Tn antigens. We discuss the effects on DC function induced throughout the engagement of MGL, highlighting the importance of the antigen structure in the modulation of immune response. Indeed modifying Tn-density, the length, and steric structure of the Tn-antigens can result in generating immunogens that can efficiently bind to MGL, strongly activate DCs, mimic the effects of a danger signal, and achieve an efficient presentation in HLA classes I and II compartments.

## 1. Introduction

Dendritic cells (DCs), as professional antigen-presenting cells (APCs), sense the microenvironment through different types of receptors to scan local environmental changes and eliminate incoming pathogens [1]. They play an essential role in the uptake of self- or pathogen-associated antigens, thus, steering and directing the immune response. After activation, DCs migrate to the draining lymph nodes, where they initiate specific immunity. DCs, similarly to macrophages (MØs) and B cells, are equipped with a set of receptors that recognize, capture, and internalize foreign antigens to facilitate an efficient processing and presentation through MHC II and I molecules. While B cells are specialized to recognize an extensive variety of epitopes due to the presence of somatically variable surface immunoglobulin receptors, DCs and MØs rely on a set of germline-encoded membrane receptors for the discrimination and recognition of antigenic determinants. Besides complement and Fc receptors, DCs express a large array of pattern-recognition receptors (PRRs), which have evolved to activate and modulate immune functions upon encountering ligands from “nonself” (pathogen-associated molecular patterns (PAMPs)), “damaged self” (damage-associated molecular patterns (DAMPs)), or “altered self” as in cancer (tumor-associated molecular patterns (TAMPs)) [2, 3]. The PRRs are a heterogeneous group of receptor subfamilies, among which the best characterized are the toll-like receptors (TLRs) and the C-type lectin receptors (CLRs). The TLRs respond to a wide variety of pathogen-derived molecular structures with a response characterized by the activation of proinflammatory signaling pathways [4]. However, TLRs are not able to internalize antigens. This function is instead largely covered by CLRs. CLRs were initially thought to function only as scavenger receptors able to bind various pathogens upon recognition of particular carbohydrate profiles through at least one carbohydrate recognition domain (CRD). CLRs recognize and internalize specific carbohydrate antigens in Ca^2+^-dependent manner [5] thus influencing the outcome of the immune response [6]. In fact, the importance of C-type lectins is highlighted by the fact that these receptors are able to trigger numerous cellular and immunological responses critical for the control and regulation of infection, homeostasis, autoimmunity, allergy, and cancer [7–9]. Several studies have demonstrated that some C-type lectins may function as adhesion, signaling, or antigen-uptake receptors [10–12]. These results are consistent with the fact that CLRs are present on MØs and DCs, which play a role in the initial step of capturing the antigens carrying carbohydrates [13]. Pathogens recognition by CLRs leads to its internalization, degradation, and subsequent antigen presentation. Besides antigen recognition and internalization, CLRs are also able to induce intracellular signaling and recruit other molecules such as TLRs that can modulate the signaling cascade [14]. In particular, CLR triggering by different pathogens can induce diverse immune responses [8]. For this reason and for their potential implication in the therapy of immune diseases and cancer, this receptor family has received great attention in recent years.

The most important molecules from the CLR family include macrophage galactose type C-type lectin (MGL), dendritic cell-specific intercellular adhesion molecule-3-grabbing nonintegrin (DC-SIGN), mannose receptor (MR), DEC205, Dectin-1, and Langerin. These receptors are able to trigger distinct signaling pathways that modulate APC functions through the expression of specific molecules and cytokines, determining the polarization of T cells [8].

**Figure 1:**
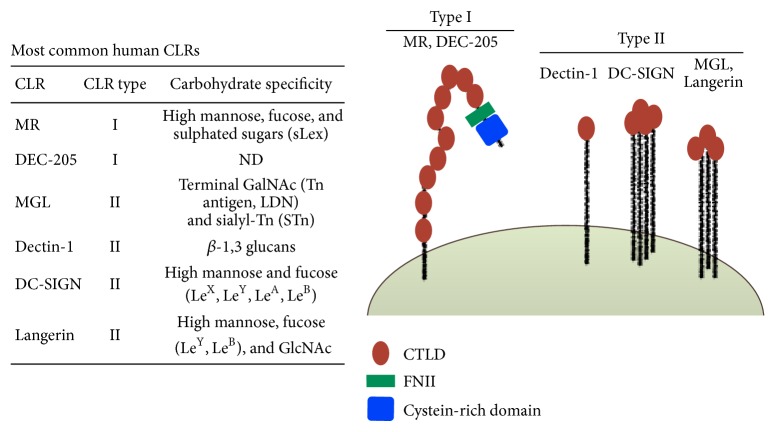
Schematic representation of type I and type II C-type lectins and lectin-like receptors. Type I CLRs (MR, DEC-205) are composed of a N-terminal cysteine-rich domain, a single fibronectin type II (FNII) domain, and 8–10 CTLDs all expressing CRDs. Type II CLRs (Langerin, DC-SIGN, MGL) or lectin-like receptors (Dectin-1) are composed of a single CTLD, an extracellular stalk region, a transmembrane region, and a N-terminal cytoplasmic tail with or without a signaling motif or proline-rich region. Langerin, DC-SIGN, MGL, and Dectin-1 express a CRD on their CTLD. CTLD, C-type lectin like domain; CLR, C-type lectin receptor; CRD, carbohydrate recognition domain; MR, mannose receptor; DC-SIGN, DC-specific ICAM3-grabbing nonintegrin; MGL, macrophage galactose type C-type lectin.

CLRs such as DC-SIGN, MGL, and Langerin are well characterized for their specificity for high-mannose, fucose-containing glycans (Lewis^A,B,X,Y^), GalNAc (N-Acetylgalactosamine) and high mannose, fucose (Lewis^Y^, Lewis^B^), and GlcNAc (N-Acetylglucosamine), respectively (Figure 1) [15, 16]. These glycan structures can be expressed by both mammalian cells and pathogens, reflecting CLR dual function in host-pathogen recognition and immune cell responses.

The APCs are the first line of defence responsible for clearing pathogens and they subsequently initiate adaptive immune responses. The DC-expressed C-type lectins mannose receptor, DC-SIGN, MGL, Dectin-1, and Langerin on Langherans cells are involved in glycan-mediated pathogen recognition and internalization of antigen for loading on MHC class I and class II molecules, thereby facilitating effective antigen-specific CD4^+^ and CD8^+^ T cell responses [17]. Thus, most C-type lectins are antigen-uptake receptors that facilitate MHC-restricted antigen presentation to T cells. All these lectins are considered powerful signaling molecules that positively or negatively instruct DC differentiation and subsequent T cell responses.

In this review, we focus on the role of human MGL as prototype receptor highly specialized in the glycan recognition in immune system, on its high plasticity and capacity to modulate the immune response conditioned by the type of the ligand.

## 2. Human MGL in Immune Response

MGL is a CLR exclusively expressed* in vivo* by human DCs of skin and lymph nodes and* in vitro* by macrophages and monocytes derived DCs [18, 19]. Within the CLR family, MGL is the only CLR within the human immune system that exclusively recognizes terminal *α*- or *β* N-acetylgalactosamine (GalNAc or Tn) residues [20, 21]. In particular, its carbohydrate recognition domains contain a QPD sequence that is responsible for recognition of GalNAc/Tn residues of* N*- and* O*-glycans carried by glycoproteins and/or glycosphingolipids of helminths, bacteria, filovirus, and tumor-associated antigens [20].

In mice, there are two homologs of human MGL, MGL1, and MGL2 [22], expressed by dermal DCs and alternatively activated macrophages [18, 23]. MGL1 is highly specific for Lewis X and Lewis A structures, while MGL2, similar to the human MGL, recognizes* N*-GalNAc and galactose and* O*-linked Tn-antigen, TF-antigen, and core 2 structures [24].

**Figure 2:**
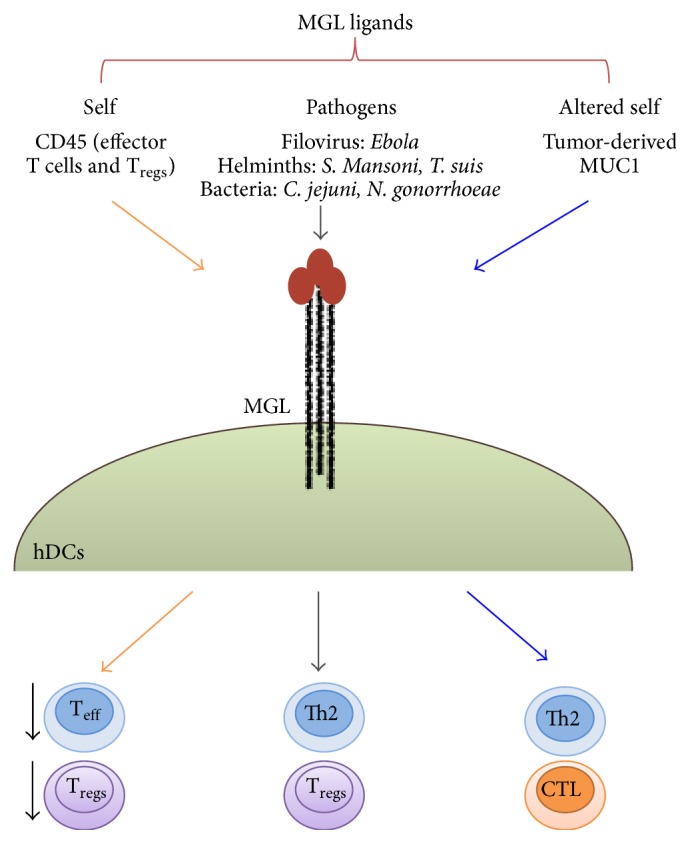
Representation of ligands binding human MGL expressed by DCs and impact of MGL triggering on immune response.

Human MGL is equipped with a partial dileucine zipper, with YENF internalization motifs [25], and is involved in (a) the recognition of a large plethora of pathogens by DCs [26, 27], (b) in the maintenance of homeostasis [28], and (c) in the interaction with TAA derived from aberrant glycosylation processes [16, 29]. More recently, our group has also demonstrated the role of MGL in immunosuppression given by its capacity to modulate regulatory T cell function [30] (Figure 2). Thus MGL acts as promiscuous receptor that can bind more than one ligand modulating various types of immune response. The pathogens that bind human MGL include* Ebola* virus through the interaction with the viral envelope protein GP2. It was demonstrated that this lectin promotes filovirus entry suggesting a role for MGL in viral replication* in vivo* [31]. Also the helminths* Schistosoma mansoni* and* Trichuris suis* interact with MGL [32, 33]. Schistosoma soluble egg antigens (SEA) consistently are inducers of Th2 responses in different experimental settings either* in vitro* or* in vivo* and both in humans or in animal models. In particular, studies performed on DCs have been instrumental in understanding the polarization of immune responses towards Th2 by SEA [32, 34–38]. While DCs fail to show classic signs of maturation when stimulated with SEA [32, 34],* in vitro* experiments show that SEA-primed monocyte-derived DCs (both human and murine) are very potent in polarizing naive Th cells towards a Th2 type [34, 35, 38].

DCs show remarkable phenotypic changes when recognizing soluble products (SPs) of* Trichuris suis*, a pig whipworm that is experimentally used in therapies to ameliorate inflammation in patients with Crohn's disease and multiple sclerosis [39].* Trichuris suis* glycans play an important role in the capacity to suppress proinflammatory cytokine and chemokine production of DCs interacting with DCs via CLRs, such as MGL. In particular,* T. suis* SPs suppress the production of the proinflammatory cytokines, IL-12, TNF-*α*, and IL-6, and many proinflammatory chemokines. These properties, in combination with upregulation of OX40L and CXCL16 expression, are regarded as positive signals for Th2 polarization [40, 41].

Human MGL, through GalNAc-terminated lipopolysaccharide (LPS) and glycoproteins, interacts also with bacteria such as* Campylobacter jejuni* and* Neisseria gonorrhoeae* [26, 27].* N. gonorrhoeae* phenotype C, carrying a terminal N-acetylgalactosamine, primarily interacted with MGL and skewed immunity towards the Th2 lineage through IL-4 production, whereas* N. gonorrhoeae* variant A with a terminal N-acetylglucosamine on its lipooligosaccharide (LOS) was recognized by DC-SIGN and induced significantly more IL-10 production.

In humans, monocyte-derived DCs express moderate MGL levels, which become negative after DC maturation. In addition variation of its expression related to seasonal changes is observed [42]. Moreover, MGL is upregulated on tolerogenic DCs generated in the presence of glucocorticoids and during chronic inflammatory conditions such as rheumatoid arthritis, implicating MGL in immune regulation [43]. In fact MGL has been shown to interact directly with a subset of CD4^+^ and/or CD8^+^ effector T cells. The MGL ligand on these T cells was identified as CD45, which exposes terminal GalNAc (Tn) structures. This interaction negatively regulates T cell receptor-mediated signaling by decreasing the phosphatase activity of CD45 and inhibiting lymphocyte protein tyrosine kinase (Lck) activation and Ca^2+^ mobilization [9] that results in enhanced apoptosis of T cells and reduced secretion of proinflammatory cytokines [9, 43]. We have more recently demonstrated that also CD45RA^+^ Treg subpopulation is affected by MGL engagement. CD45RA-MGL cross-linking induces a decrease of Treg immunosuppressive activity by affecting CD45RA and TCR signaling and an increase of Foxp3 methylation accompanied by a reduced production of suppressive cytokines [30]. Recent evidence indicates that human MGL expressed on DCs is also able to generate antigen specific IL-10 producing CD4^+^ T cells when stimulated with foreign and self-antigens fused to an anti-MGL antibody [44].

These results demonstrate an important function for MGL in the regulation of T cell homeostasis and in the silencing of potentially harmful T cell activation.

## 3. MGL in Cancer Immunity

The capacity of CLRs to bind, process, and cross-present antigens has received much attention in the field of cancer immunity. Several of tumor-related glycoforms of self-antigens are in fact specific ligands for CLRs expressed on DCs, such as MGL. These evidences open up a new area of research to investigate whether these tumor specific glycoforms affect CLR signaling and DC differentiation, thereby modulating innate and adaptive antitumor response. MGL is able to recognize the mucin MUC1, an* O*-linked glycosylated transmembrane protein normally expressed on the apical surface of epithelial cells, but aberrantly expressed in a broad spectrum of carcinomas. Upon malignant transformation, MUC1 loses polarity and becomes overexpressed and aberrantly glycosylated, revealing an immunogenic region of tandem repeats of 20 residues. The novel MUC1 glycoforms that arise carry shortened glycan moieties: Tn (GalNAc), T (Gal*β*1, 3GalNAc), ST (NeuAc*α*2, 3Gal*β*1, and 3GalNAc), and STn (NeuAc*α*2, 6GalNAc) [20, 45]. Because MGL recognizes GalNAc-containing epitopes frequently expressed on the surface of cancer cells and is involved in the regulation of the adaptive and innate immune response, it was chosen as a probe for glycoprofiling in breast cancer. Results indicate that high MGL-binding molecules in breast were associated with the expression of HER2/neu [46, 47]. In particular, it was demonstrate that detection of Tn ligands in mammary tissue is feasible employing the MGL recombinant protein and that this experimental approach permits recognizing posttranslational modifications (PTMs) such as phosphorylation and glycosylation. The identification of tumor-associated glycans, which potentially interact with the CRDs, could become a tool to identify those patients who will profit from MGL based specific therapeutic approaches. Tn antigen has previously been associated with worse survival [48, 49] and recent research indicates that tumor-specific Tn expression not only promotes tumor cell invasiveness [50] but also alters the immunogenicity of tumor antigens [51]. MGL is able to distinguish healthy tissue from tumor through its specific recognition of Tn antigen.* In vitro* studies, using CRC cell lines, showed an association between MGL ligand expression and the presence of BRAFV600E, suggesting a model in which activating BRAF mutations, and possibly other oncogenic alterations that activate the MAPK pathway, lead to an altered tumor cell glycosylation profile and enhanced expression of MGL ligands [48]. These aberrant glycans on tumor cells may have the ability to suppress antitumor immune responses through activation of the MGL receptor on DC. In fact, the prognostic value of MGL-binding to tumor cells is predominantly evident in stage III of colon cancer patients and not in stage II patients, that is, when tumor cells are no longer confined to the intestine but have spread into the local lymph nodes.

Recently MGL has been shown to bind also STn [21, 52, 53]. Although the binding of glycoproteins carrying Tn has been investigated [16, 54], the actual role of STn-carrying proteins binding to MGL is still to be fully investigated. Highly purified recombinant human MUC1 glycoproteins and MUC1 glycopeptides carrying either Tn or STn glycans bind MGL expressed by immature monocyte-derived DCs and by K562 transfected with MGL [52]. The interaction with the two glycoforms displays a similar affinity as demonstrated by atomic force microscopy (AFM). This is important because, although the vast majority of breast cancers stain for Tn [50], the 20–25% of breast cancer express the STn glycoforms on plasma membrane [54].

The most relevant result in tumor is the interaction between MGL, expressed by DCs, and tumor through the Tn glycans expressed by MUC1 tumor associated antigen [16, 29, 55]. The signaling activated by MGL has been recently well characterized in DCs. The MGL engagement with an anti-MGL antibody or MUC1-Tn glycopeptide (60 amino acids) triggers the extracellular signal-regulated kinases 1 and 2 (ERK1,2) and nuclear factor-*κ*B (NF-*κ*B) pathways and induces phenotypic and functional DC maturation to license DCs to initiate a strong CD8^+^ T cell immune response [55]. Moreover, similar to other CLRs, MGL signaling synergies with TLR2-induced pathways in DCs, leading to elevated IL-10 mRNA levels and enhanced TNF-*α* mRNA stability. In addition, MGL signaling promoted phosphorylation of the MAPK ERK1,2 and the transcription factor CREB. At the same time, NF-*κ*B seems to be crucial for the IL-10 response and dispensable for TNF-*α* production. Together, these results demonstrate that MGL activation modulates DC maturation and this ability highlights the possibility to use this receptor as a target for anticancer vaccination strategies.

## 4. The Nature of the Ligand Can Modulate Signals through MGL

The coupling of CLRs to different signal transduction modules is influenced not only by the receptor but also by the nature, density, size, and architecture of the ligand, which can affect the rate of receptor internalization and trafficking to different intracellular compartments. Understanding how the variety of ligands can trigger differential CLR signaling and function represents a fascinating biological challenge.

**Figure 3:**
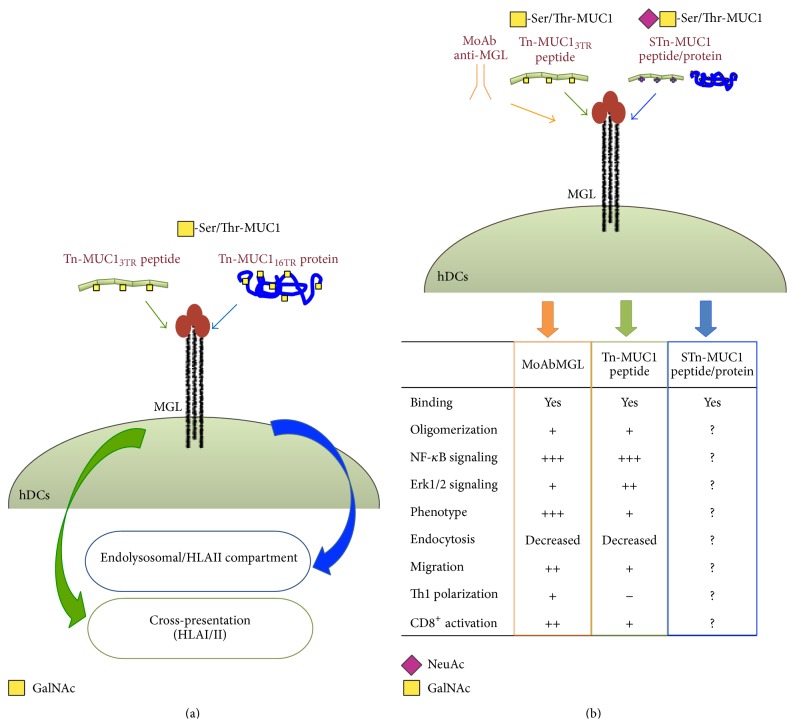
The structure of tumor antigen formulations internalized by MGL influences their processing and DC performance. (a) Recombinant Tn-MUC1_16TR_ protein remains blocked in DCs HLA class II compartment after internalization through MGL, while the shorter Tn-MUC1_3TR_ peptide (60 amino acids) is processed in HLA classes I and II compartments. (b) Effects induced on DC functions by MGL engagement with MoAb anti-MGL and Tn-MUC1 peptide.

MGL was first described as a C-Type lectin with high endocytic activity regulated by the “YENF” consensus sequence contained in the cytoplasmic tail. MGL mediated endocytosis of soluble Tn-carrying antigens resulted in processing in HLAII compartment and activation of antigen specific CD4^+^ T cells [25, 56]. However these reports did not investigate the processing in HLAI compartment and the possible induction of CD8^+^ T cells responses. The effects on DCs induced by MGL engagement differ on the basis of ligands. Employing the Tn-MUC1 glycoform as cancer model glycoantigen, we showed that the structure of the MGL ligand dictates the upcoming intracellular processing. In fact, upon MGL engagement, the large soluble recombinant Tn-MUC1 glycoprotein (Tn-MUC1_16TR_) corresponding to the one shed* in vivo* by epithelial cancers remained trapped in the endolysosomal/HLAII compartment, while the MUC1 peptide (Tn-MUC1_3TR_), 60 amino acids long, carrying 9 Tn moles colocalized both in HLAII and HLAI compartments [16, 57] (Figure 3(a)).

It is interesting to note that this distinct intracellular routing of the two MUC1 based ligands matches with a distinct signaling and phenotypic profile of DCs induced by MGL engagement.

In fact, cross-processing in HLAI compartment of the 9Tn-MUC1_3TR_ is accompanied by triggering of ERK1,2 phosphorylation, activation of NF-*κ*B signal, and upregulation of maturative markers. These effects are similar, although weaker, to the ones induced by a strong ligand as a specific anti-MGL MoAb [55], suggesting that MGL engagement could be used as adjuvant in DC-based vaccination (Figure 3(b)). On the other hand, preliminary and unpublished results from our laboratory indicate that the MGL mediated endocytosis of the large soluble Tn-MUC1 molecule, retained in HLAII compartment, does not modify the balance of ERK1,2/NFkB and DC phenotype. How the different structure of the ligand and therefore its avidity and affinity can modulate intracellular pathway activated by MGL cytoplasmic tail is yet to be defined. For other C-type lectins it has been proposed that ligand affinity and avidity as well as the particulate form of the ligand are crucial for the clustering of the receptor and the generation of the “endocytic synapsis.” These steps seem to be important for tuning the strength of the signaling and determining the type of immunological response induced [58].

MGL oligomerization appears to occur independently by the structure of its ligand; however signaling and phenotypic changes can be different. It has shown that heterodimerization with other receptors and association with distinct PPRs modulate and enhance pathogen sensor function of several CLRs. MGL has been shown to synergize with TLR2-induced pathways in a study employing artificial model ligands, not present in the physiological microenvironment [59]. The working hypothesis that is sketched out by these evidences is that the ligand structure is not an absolute requirement for MGL oligomerization since this event appears to be independent of the nature of the ligand (peptide, protein, or single carbohydrate). However the structure becomes relevant for the functional outcome. The recruitment of alternative array of adaptor molecules at the “endocytic synapsis” may be involved in the differential intracellular sorting of the ligand and in the tuning of the signaling pathways crucial for DC polarization.

For this reason, it will be interesting to further characterize the interaction between MGL and its ligand, in terms of affinity, avidity, and ligand structure in order to design molecules to be employed as immunomodulators in therapeutic strategies for several pathologies, as well as for cancer.

## 5. Conclusions

The study of MGL and its role in DCs as well as in macrophage functions is an open interesting area of research. The specificity for terminal GalNAc residues combined with the restricted tissue expression of this carbohydrate residue makes MGL a very specific detector of pathogens and a highly sensitive modulator of APCs in physiological as well as in inflammatory and cancer microenvironment.

The engagement of MGL by GalNAc carrying structures induces oligomerization of the receptor and internalization of the ligand; however the intracellular signaling pathways triggered can profoundly vary depending on the structure of the ligands, differentially affecting function of DCs and the resulting immune response.

In this view, the possibility to exploit MGL targeting as a way to modulate APC functions is an appealing hypothesis for the design of immunotherapeutic intervention.

Avidity and affinity of MGL ligand can be modulated by modifying Tn-density, the length, and steric structure of the backbone peptides. For cancer therapy, activation of DCs could be obtained by mimicking the effects of a danger signal and achieving an efficient presentation in HLA classes I and II pathways. Moreover, a wide variety of immunogens carrying Tn-epitopes could be envisaged, where the Tn-peptide stretch could only be a way to deliver other TAAs as well as other molecules. On the other hand, the fine-tuning of MGL function could lead to the induction of “Th2 oriented DCs” and interfere in the immunosuppressive/inflammatory network induced by pathogens infection. So far, investigators have focused their attention on the understanding the effect of MGL engagement on DCs. However, one has to bear in mind that the MGL-GalNAc ligand engagement mediates a one to one interaction between two cells. Thus functional changes may also occur in the cell carrying the GalNAc moieties. This mechanism seems to be particularly relevant in the physiological tissue homeostasis such as in T cell compartment. In this prospective, the design of optimal immunotherapeutic molecules based on MGL triggering could be novel approaches for the treatment of autoimmune diseases.

It is also important to retain that the molecular targeting and the choice of the antigen represent only “one side of the coin” in designing immunotherapeutic approaches. The choice of the optimal DC subset for priming T cells and strategies to contain or eliminate immunosuppression are other crucial parameters that should be considered to obtain an efficacious and long-lasting immune response.

## References

[1] Pulendran B., Palucka K., Banchereau J. (2001). Sensing pathogens and tuning immune responses. *Science*.

[2] Janeway C. A., Medzhitov R. (2002). Innate immune recognition. *Annual Review of Immunology*.

[3] Dambuza I. M., Brown G. D. (2015). C-type lectins in immunity: recent developments. *Current Opinion in Immunology*.

[4] Kawai T., Akira S. (2008). Toll-like receptor and RIG-1-like receptor signaling. *Annals of the New York Academy of Sciences*.

[5] Zelensky A. N., Gready J. E. (2005). The C-type lectin-like domain superfamily. *The FEBS Journal*.

[6] Van Kooyk Y., Geijtenbeek T. B. H. (2003). DC-SIGN: escape mechanism for pathogens. *Nature Reviews Immunology*.

[7] Engering A., Geijtenbeek T. B. H., Van Vliet S. J. (2002). The dendritic cell-specific adhesion receptor DC-SIGN internalizes antigen for presentation to T cells. *The Journal of Immunology*.

[8] Geijtenbeek T. B. H., Gringhuis S. I. (2009). Signalling through C-type lectin receptors: shaping immune responses. *Nature Reviews Immunology*.

[9] van Vliet S. J., García-Vallejo J. J., van Kooyk Y. (2008). Dendritic cells and C-type lectin receptors: coupling innate to adaptive immune responses. *Immunology and Cell Biology*.

[10] Brown G. D., Herre J., Williams D. L., Willment J. A., Marshall A. S. J., Gordon S. (2003). Dectin-1 mediates the biological effects of *β*-glucans. *Journal of Experimental Medicine*.

[11] Gantner B. N., Simmons R. M., Canavera S. J., Akira S., Underhill D. M. (2003). Collaborative induction of inflammatory responses by dectin-1 and Toll-like receptor 2. *Journal of Experimental Medicine*.

[12] Chakraborty P., Ghosh D., Basu M. K. (2001). Modulation of macrophage mannose receptor affects the uptake of virulent and avirulent *Leishmania donovani* promastigotes. *Journal of Parasitology*.

[13] Oo-puthinan S., Maenuma K., Sakakura M. (2008). The amino acids involved in the distinct carbohydrate specificities between macrophage galactose-type C-type lectins 1 and 2 (CD301a and b) of mice. *Biochimica et Biophysica Acta (BBA): General Subjects*.

[14] O'Neill L. A. J. (2008). When signaling pathways collide: positive and negative regulation of toll-like receptor signal transduction. *Immunity*.

[15] van Vliet S. J., Saeland E., van Kooyk Y. (2008). Sweet preferences of MGL: carbohydrate specificity and function. *Trends in Immunology*.

[16] Napoletano C., Rughetti A., Agervig Tarp M. P. (2007). Tumor-associated Tn-MUC1 glycoform is internalized through the macrophage galactose-type C-type lectin and delivered to the HLA class I and II compartments in dendritic cells. *Cancer Research*.

[17] Tacken P. J., de Vries I. J. M., Gijzen K. (2005). Effective induction of naive and recall T-cell responses by targeting antigen to human dendritic cells via a humanized anti-DC-SIGN antibody. *Blood*.

[18] Raes G., Brys L., Dahal B. K. (2005). Macrophage galactose-type C-type lectins as novel markers for alternatively activated macrophages elicited by parasitic infections and allergic airway inflammation. *Journal of Leukocyte Biology*.

[19] Van Vliet S. J., Paessens L. C., Broks-van Den Berg V. C. M., Geijtenbeek T. B. H., Van Kooyk Y. (2008). The C-type lectin macrophage galactose-type lectin impedes migration of immature APCs. *The Journal of Immunology*.

[20] Van Vliet S. J., Van Liempt E., Saeland E. (2005). Carbohydrate profiling reveals a distinctive role for the C-type lectin MGL in the recognition of helminth parasites and tumor antigens by dendritic cells. *International Immunology*.

[21] Mortezai N., Behnken H. N., Kurze A.-K. (2013). Tumor-associated Neu5Ac-Tn and Neu5Gc-Tn antigens bind to C-type lectin CLEC10A (CD301, MGL). *Glycobiology*.

[22] Tsuiji M., Fujimori M., Ohashi Y. (2002). Molecular cloning and characterization of a novel mouse macrophage C-type lectin, mMGL2, which has a distinct carbohydrate specificity from mMGL1. *The Journal of Biological Chemistry*.

[23] Kumamoto Y., Denda-Nagai K., Aida S., Higashi N., Irimura T. (2009). MGL2^+^ dermal dendritic cells are sufficient to initiate contact hypersensitivity in vivo. *PLoS ONE*.

[24] Singh S. K., Streng-Ouwehand I., Litjens M. (2009). Characterization of murine MGL1 and MGL2 C-type lectins: distinct glycan specificities and tumor binding properties. *Molecular Immunology*.

[25] Van Vliet S. J., Aarnoudse C. A., Broks-van den Berg V. C. M., Boks M., Geijtenbeek T. B. H., van Kooyk Y. (2007). MGL-mediated internalization and antigen presentation by dendritic cells: a role for tyrosine-5. *European Journal of Immunology*.

[26] van Sorge N. M., Bleumink N. M. C., van Vliet S. J. (2009). *N*-glycosylated proteins and distinct lipooligosaccharide glycoforms of Campylobacter jejuni target the human C-type lectin receptor MGL. *Cellular Microbiology*.

[27] Van Vliet S. J., Steeghs L., Bruijns S. C. M. (2009). Variation of *Neisseria gonorrhoeae* lipooligosaccharide directs dendritic cell-induced T helper responses. *PLoS Pathogens*.

[28] Van Vliet S. J., Gringhuis S. I., Geijtenbeek T. B. H., Van Kooyk Y. (2006). Regulation of effector T cells by antigen-presenting cells via interaction of the C-type lectin MGL with CD45. *Nature Immunology*.

[29] Saeland E., Van Vliet S. J., Bäckström M. (2007). The C-type lectin MGL expressed by dendritic cells detects glycan changes on MUC1 in colon carcinoma. *Cancer Immunology, Immunotherapy*.

[30] Zizzari I. G., Martufi P., Battisti F. (2015). The macrophage galactose-type C-type lectin (MGL) modulates regulatory T cell functions. *PLoS ONE*.

[31] Takada A., Fujioka K., Tsuiji M. (2004). Human macrophage C-type lectin specific for galactose and N-acetylgalactosamine promotes filovirus entry. *Journal of Virology*.

[32] Van Liempt E., Van Vliet S. J., Engering A. (2007). Schistosoma mansoni soluble egg antigens are internalized by human dendritic cells through multiple C-type lectins and suppress TLR-induced dendritic cell activation. *Molecular Immunology*.

[33] Meevissen M. H. J., Driessen N. N., Smits H. H. (2012). Specific glycan elements determine differential binding of individual egg glycoproteins of the human parasite *Schistosoma mansoni* by host C-type lectin receptors. *International Journal for Parasitology*.

[34] MacDonald A. S., Straw A. D., Bauman B., Pearce E. J. (2001). CD8- dendritic cell activation status plays an integral role in influencing Th2 response development. *The Journal of Immunology*.

[35] De Jong E. C., Vieira P. L., Kalinski P. (2002). Microbial compounds selectively induce Th1 cell-promoting or Th2 cell-promoting dendritic cells in vitro with diverse th cell-polarizing signals. *The Journal of Immunology*.

[36] Kane C. M., Cervi L., Sun J. (2004). Helminth antigens modulate TLR-initiated dendritic cell activation. *The Journal of Immunology*.

[37] Cervi L., MacDonald A. S., Kane C., Dzierszinski F., Pearce E. J. (2004). Cutting edge: dendritic cells copulsed with microbial and helminth antigens undergo modified maturation, segregate the antigens to distinct intracellular compartments, and concurrently induce microbe-specific Th1 and helminth-specific Th2 responses. *Journal of Immunology*.

[38] Jankovic D., Kullberg M. C., Caspar P., Sher A. (2004). Parasite-induced Th2 polarization is associated with down-regulated dendritic cell responsiveness to Th1 stimuli and a transient delay in T lymphocyte cycling. *The Journal of Immunology*.

[39] Klaver E. J., Kuijk L. M., Laan L. C. (2013). *Trichuris suis*-induced modulation of human dendritic cell function is glycan-mediated. *International Journal for Parasitology*.

[40] Jenkins S. J., Perona-Wright G., Worsley A. G. F., Ishii N., MacDonald A. S. (2007). Dendritic cell expression of OX40 ligand acts as a costimulatory, not polarizing, signal for optimal Th2 priming and memory induction in vivo. *Journal of Immunology*.

[41] Bruckner M., Dickel D., Singer E., Legler D. F. (2012). Converse regulation of CCR7-driven human dendritic cell migration by prostaglandin E_2_ and liver X receptor activation. *European Journal of Immunology*.

[42] Zizzari I. G., Napoletano C., Rughetti A., Rahimi H., Nuti M. (2013). Seasonal modulation of the C-type lectin MGL on human DCs. *Open Journal of Immunology*.

[43] Van Vliet S. J., Van Liempt E., Geijtenbeek T. B. H., Van Kooyk Y. (2006). Differential regulation of C-type lectin expression on tolerogenic dendritic cell subsets. *Immunobiology*.

[44] Li D., Romain G., Flamar A.-L. (2012). Targeting self- and foreign antigens to dendritic cells via DC-ASGPR generates IL-10-producing suppressive CD4^+^ T cells. *Journal of Experimental Medicine*.

[45] Burchell J. M., Mungul A., Taylor-Papadimitriou J. (2001). O-linked glycosylation in the mammary gland: changes that occur during malignancy. *Journal of Mammary Gland Biology and Neoplasia*.

[46] Welinder C., Baldetorp B., Borrebaeck C., Fredlund B.-M., Jansson B. (2011). A new murine IgG1 anti-Tn monoclonal antibody with in vivo anti-tumor activity. *Glycobiology*.

[47] Nollau P., Wolters-Eisfeld G., Mortezai N. (2013). Protein domain histochemistry (PDH): binding of the carbohydrate recognition domain (CRD) of recombinant human glycoreceptor CLEC10A (CD301) to formalin-fixed, paraffin embedded breast cancer tissues. *Journal of Histochemistry and Cytochemistry*.

[48] Lenos K., Goos J. A., Vuist I. M. (2015). MGL ligand expression is correlated to BRAF mutation and associated with poor survival of stage III colon cancer patients. *Oncotarget*.

[49] Itzkowitz S. H., Yuan M., Montgomery C. K. (1989). Expression of Tn, sialosyl-Tn, and T antigens in human colon cancer. *Cancer Research*.

[50] Gill D. J., Tham K. M., Chia J. (2013). Initiation of GalNAc-type O-glycosylation in the endoplasmic reticulum promotes cancer cell invasiveness. *Proceedings of the National Academy of Sciences of the United States of America*.

[51] Freire T., Lo-Man R., Bay S., Leclerc C. (2011). Tn glycosylation of the MUC6 protein modulates its immunogenicity and promotes the induction of Th17-biased T cell responses. *The Journal of Biological Chemistry*.

[52] Beatson R., Maurstad G., Picco G. (2015). The breast cancer-associated glycoforms of MUC1, MUC1-Tn and sialyl-Tn, are expressed in *COSMC* wild-type cells and bind the C-type lectin MGL. *PLoS ONE*.

[53] Jégouzo S. A., Quintero-Martínez A., Ouyang X., Dos Santos Á., Taylor M. E., Drickamer K. (2013). Organization of the extracellular portion of the macrophage galactose receptor: a trimeric cluster of simple binding sites for *N*-acetylgalactosamine. *Glycobiology*.

[54] Miles D. W., Happerfield L. C., Smith P. (1994). Expression of sialyl-Tn predicts the effect of adjuvant chemotherapy in node-positive breast cancer. *British Journal of Cancer*.

[55] Napoletano C., Zizzari I. G., Rughetti A. (2012). Targeting of macrophage galactose-type C-type lectin (MGL) induces DC signaling and activation. *European Journal of Immunology*.

[56] Higashi N., Fujioka K., Denda-Nagai K. (2002). The macrophage C-type lectin specific for galactose/*N*-acetylgalactosamine is an endocytic receptor expressed on monocyte-derived immature dendritic cells. *Journal of Biological Chemistry*.

[57] Rughetti A., Rahimi H., Belleudi F. (2014). Microvesicle cargo of tumor-associated MUC1 to dendritic cells allows cross-presentation and specific carbohydrate processing. *Cancer Immunology Research*.

[58] Iborra S., Sancho D. (2015). Signalling versatility following self and non-self sensing by myeloid C-type lectin receptors. *Immunobiology*.

[59] van Vliet S. J., Bay S., Vuist I. M. (2013). MGL signaling augments TLR2-mediated responses for enhanced IL-10 and TNF-*α* secretion. *Journal of Leukocyte Biology*.

